# Bmp-12 activates tenogenic pathway in human adipose stem cells and affects their immunomodulatory and secretory properties

**DOI:** 10.1186/s12860-017-0129-9

**Published:** 2017-02-18

**Authors:** Weronika Zarychta-Wiśniewska, Anna Burdzinska, Agnieszka Kulesza, Kamila Gala, Beata Kaleta, Katarzyna Zielniok, Katarzyna Siennicka, Marek Sabat, Leszek Paczek

**Affiliations:** 1 Department of Immunology, Transplantology and Internal Medicine, Transplantation Institute, Medical University of Warsaw, Nowogrodzka str. 59, 02-006 Warsaw, Poland; 2 Department of Clinical Immunology, Transplantation Institute, Medical University of Warsaw, Warsaw, Poland; 3 Department of Physiological Sciences, Faculty of Veterinary Medicine, Warsaw University of Life Sciences, Warsaw, Poland; 4 Department of Regenerative Medicine, Maria Sklodowska-Curie Memorial Cancer Center, Warsaw, Poland; 5 Department of Bioinformatics, Institute of Biochemistry and Biophysics, Polish Academy of Sciences, Warsaw, Poland

**Keywords:** Adipose stem cells, Mesenchymal stem cells, BMP-12, Tenogenic differentiation, Secretory activity

## Abstract

**Background:**

Cell-based therapy is a treatment method in tendon injuries. Bone morphogenic protein 12 (BMP-12) possesses tenogenic activity and was proposed as a differentiating factor for stem cells directed to transplantation. However, BMPs belong to pleiotropic TGF-β superfamily and have diverse effect on cells. Therefore, the aim of this study was to determine if BMP-12 induces tenogenic differentiation of human adipose stem cells (hASCs) and how it affects other features of this population.

**Results:**

Human ASCs from 6 healthy donors were treated or not with BMP-12 (50 or 100 ng/ml, 7 days) and tested for gene expression (*COLL1, SCX, MKH, DCN, TNC, RUNX2*), protein expression (COLL1, COLL3, MKH), proliferation, migration, secretory activity, immunomodulatory properties and susceptibility to oxidative stress. RT-PCR revealed up-regulation of *SCX, MKH* and *RUNX2* genes in BMP-12 treated cells (2.05, 2.65 and 1.87 fold in comparison to control, respectively, *p* < 0.05) and Western Blot revealed significant increase of COLL1 and MHK expression after BMP-12 treatment. Addition of BMP-12 significantly enhanced secretion of VEGF, IL-6, MMP-1 and MPP-8 by hASCs while had no effect on TGF-β, IL-10, EGF and MMP-13. Moreover, BMP-12 presence in medium attenuated inhibitory effect of hASCs on allo-activated lymphocytes proliferation. At the same time BMP-12 displayed no influence on hASCs proliferation, migration and susceptibility to oxidative stress.

**Conclusion:**

BMP-12 activates tenogenic pathway in hASCs but also affects secretory activity and impairs immunomodulatory potential of this population that can influence the clinical outcome after cell transplantation.

**Electronic supplementary material:**

The online version of this article (doi:10.1186/s12860-017-0129-9) contains supplementary material, which is available to authorized users.

## Background

Tendon injuries are common musculoskeletal disorders that clinicians address daily, however, the question of optimal treatment is still unanswered. Naturally occurring tendon healing is far from satisfactory, because tendons after injury rarely return to their full mechanical strength [1]. Therefore, trials to improve tendon recovery by local supplementation of different bioactive components are undertaken [2]. One of the proposed approaches is to implement cellular therapy into treatment protocols both for acute tendon injuries and for chronic tendinopathies [3]. Mesenchymal stem cells (MSCs) are considered as the most promising population in those applications because of several reasons: 1) they can undergo tenogenic differentiation [4], 2) they are suitable for both autologous and allogeneic transplantation, 3) they possess immunomodulatory properties [5], 4) they stimulate tissue regeneration via paracrine effect [6]. Another concept to accelerate tendon healing is to use local delivery of growth factors which have tenogenic activity. At present, the most often proposed candidate for such a treatment is a group of highly conserved bone morphogenic proteins (BMPs): BMP-12, BMP-13 and BMP-14 (also called growth and differentiation factor 7 (GDF-7), GDF-6 and GDF-5, respectively) [7]. It was originally reported that subcutaneous or intramuscular administration of mentioned BMPs resulted in ectopic formation of tendon-like structure in contrast to BMP-2 which is well known osteogenic inductor [8]. Later, it was demonstrated that delivery of BMP-12,-13,-14 (either in form of a gene or a protein) into experimentally injured tendon improved healing parameters i.e., tensile strength [9–11]. Finally, it was shown that these BMPs, especially BMP-12, are able to activate tenogenic pathway in rat and human bone marrow (BM) derived MSCs [4, 12, 13]. Thus, based on previously published studies, the idea of using BMP-12 (-13, -14) treated MSCs or co-administration of MSCs and discussed BMPs seems to be a promising approach for tendon injuries treatment. Recently, much attention is focused on adipose derived MSCs, which are also called adipose-derived stem cells or adipose stem (stromal) cells (ASCs). They constitute attractive alternative to BM-MSCs as were demonstrated to share most important features with bone marrow counterparts. Moreover, fat harvesting is a less invasive procedure than bone marrow aspiration. However, recently it was shown in rat cells, that adipose derived MSCs possess weaker tenogenic activity than bone marrow derived counterparts [4]. Therefore, the primary aim of the present study was to evaluate if BMP-12 activate tenogenic pathway in human ASCs. However, the MSCs (ASCs) differentiation potential is only one of several clinically beneficial features of these cell populations. BMPs belong to the TGF-β superfamily and are pleiotropic molecules involved in regulation of multiply fundamental cellular functions [14]. Moreover, BMP signals influence various kinds of stem cells with very diverse outcomes [15]. Although the tenogenic activity of BMP-12 was previously studied and demonstrated [4, 16], it is not clear how this cytokine influence on other MSCs (ASCs) features. Therefore, we aimed to extend current knowledge and the secondary goal of this study was to determine whether treatment of hASCs with BMP-12 affects other potentially beneficial cells traits like proliferative and migrative capacity, secretory activity, immunomodulatory properties and susceptibility to oxidative stress.

## Methods

### Human adipose stem cells (hASCs) - isolation and identification

Adipose tissue was collected from healthy donors by liposuction. Donors were informed and agreed to participate in this study. The procedure was approved by the Local Bioethics Committee. In order to remove red blood cells, 400 ml fat tissue was mixed 2:1 vol/vol with buffered physiological salt solution (Phosphate-buffered saline- PBS) and shaken every 15 min. Following phases separation, PBS with red blood cells were discarded. Purification process was repeated three times. Afterwards, 0.075% collagenase solution from Clostridium histolyticum (Sigma – Aldrich) in PBS was added to adipose tissue (1:2 vol/vol), shaken and incubated in temp. 37 °C for 1.5 h in order to digest the tissue. The fat and collagenase mixture were shaken every 15 min. After obtaining a homogenous suspension, human albumin (20% concentration) was added (final concentration 2%) to stop the digestion reaction. Mixture was centrifuged (400 *g*) for 10 min at room temperature (RT). The liquid fat and salt interphases were discarded and the cell pellet was suspended in PBS. Cell suspension was filtered through 100 μm nylon filter, washed in PBS and centrifuged (300 *g*) for 10 min, RT. The amount and viability of the cells were determined and cells were seeded into plastic flasks at a density of 8 × 10^4^ cells/cm^2^ for further cultured in growth medium (GM) composed of DMEM-LG (Dulbecco’s modified Eagle’s Medium with low glucose; Sigma-Aldrich) supplemented with fetal calf serum (FCS; 15%; Invitrogen) and antibiotic–antimycotic solution (Penicillin-streptomycin-amfoterycin; 1.5%; Invitrogen) and incubated under standard cell culture conditions (37 °C, 5% CO_2_, 95% humidity). When primary cultures reached subconfluency, cells were detached by exposure to trypsin (0.25% trypsin with 1 mM EDTA; Invitrogen) and replated at a density of 5.0 × 10^3^ cells/cm^2^ for subsequent passage. After passage 3 cells were identified by flow cytometry and multilineage differentiation capacity and were frozen in liquid nitrogen. For experiments refrozen hASCs from 6 independent donors at passage 4–7 were used.

#### *In vitro* osteogenic differentiation

Osteogenic differentiation was performed at the third passage. Cells were cultured in hMSC Osteogenic Differentiation BulletKit™ Medium (Lonza) for 3 weeks. The medium was changed every 3 days. Osteogenic differentiation was characterized by identification of mineral depositions in extracellular matrix. At 3 weeks, the plated cells were fixed for 15 min with 4% formaldehyde and stained with Alizarin Red (Sigma-Aldrich). After staining, the wells were rinsed with distilled water and visualized by standard light microscopy.

#### *In vitro* adipogenic differentiation

Adipogenic differentiation was performed at the third passage. Cells were cultured in hMSC Adipogenic Differentiation BulletKit™ Medium (Lonza) for 3 weeks. Adipogenic differentiation was assessed using Oil Red O (Sigma-Aldrich) stain as an indicator of intracellular lipid accumulation. Prior to staining, plastic-adherent cells were fixed for 45 min with 10% formaldehyde and then for 5 min with 60% isopropanol. After fixation and staining, the wells were rinsed with distilled water and visualized by standard light microscopy.

#### *In vitro* chondrogenic differentiation

To induce chondrogenic differentiation, three-dimensional pellet culture was performed. In a 15 ml tube, 3 × 10^5^ cells were pelleted by centrifugation. Unsuspended cell pellets were cultured for 19 days in chondrogenic medium (Lonza) composed of basic medium supplemented with dexamethasone, ascorbate, ITS + supplement, pyruvate, proline, GA-1000, L-glutamine and recombinant human transforming growth factor-β3. For histological analysis, pellets were immersed in paraffin, sectioned and stained with Masson trichrome method.

#### Flow cytometry analysis

The surface antigen profiles of adipose derived MSCs at the third passage were characterized by flow cytometry. A total of 2,5 × 10^6^ cells were incubated with the following phycoerythrin (PE)-conjugated anti-mouse antibodies: CD29, CD34, CD45, CD73, CD90 and CD105 (Becton Dickinson) for 30 min, RT in the dark. Nonspecific PE-conjugated IgG was substituted as an isotype control. The fluorescence intensity of cells was evaluated using BD FACScalibur flow cytometer equipped with CellQuest Pro software (Becton Dickinson).

### Study design

Cells were grown in Petri dishes (Ø 3.5, 6 or 10 cm, depending on the experiment). At 80% confluence cells were exposed to growth medium supplemented with human recombinant BMP-12 (Sigma-Aldrich, SRP4572) in the concentrations of 50 ng/ml and/or 100 ng/ml (depending on the test). Cells from the same donors cultured at the same time in standard GM without BMP-12 served as a control. Media were changed every 2 or 3 days. After 7 days cells were harvested by trypsinisation, counted and directed either to RNA/protein isolation, or to functional tests on microplates (proliferation, migration, oxidative stress susceptibility, mixed lymphocyte reaction). If certain test required further culturing, the medium containing or not BMP-12 was used respectively. Experiments were always conducted on cells from each donor separately. The cells from different donors were not pooled in this study. This approach allowed for detection inter-individual variations. Unless it stated differently, all experiments were performed on cells from 6 different donors *n* = 6. The scheme of study design is presented in Additional file 1.

### RNA isolation

For gene expression analysis cells were treated for 7 days with/without 100 ng/ml of BMP-12. At least 3 × 10^5^ cells were used for this procedure. Isolation of total RNA was performed using RNeasy Mini Kit (Qiagen) according to the manufacturer’s instructions. RNA concentration and purity was assessed by spectrophotometer at 260 nm using NanoDrop (ND-1000 Spectrophotometer, NanoDrop Technologies, Inc).

### Real-time PCR analysis

Real-Time PCR was performed on ABI Prism 7500 Sequence Detection System using TaqMan® RNA-to-C_T_™ *1-Step* Kit (Applied Biosystems, Foster City, USA). Specific primer and probe set was purchased from Applied Biosystems: Collagen, type I, alpha 1 (Col1α1) Hs00164004_m1, Scleraxis (SCX) Hs03054634_g1, Mohawk homeobox (MKX) Hs00543190_m1, Tenascin (TNC) Hs01115665_m1, Decorin (DCN) Hs00370385_m1, Runt-related transcription factor 2 (RunX) Hs01047973_m1,. GAPDH (4333764 T) gene was used for normalization. Duplicates of each sample were performed. The relative expression of mRNA expression was calculated by 2^−ΔΔCt^ method. The result was presented as a fold change of gene expression in relation to the calibrator. Statistical analysis was performed by comparison of dCt values using non-parametric test for related data (control versus treated cells from the same population).

### Immunocytochemistry (ICC)

To assess the effect of BMP-12 treatment on expression of collagen type I and type III ICC staining was performed. For this analysis cells were seeded on Nunc™ Lab-Tek™ II CC2™ 8-Chamber Slide System. First, cells were cultured for 7 day with or without 50 or 100 ng/ml BMP-12. For ICC quantification, the incubation time of was shortened to 5 days in order to avoid full confluence which would hinder subsequent analysis). At the end of experiment, hASCs were fixed with 4% paraformaldehyde (10 min, RT), permeabilized with 70% methanol (15 min, -20 °C), treated with blocking solution composed of 5% normal donkey serum, 1% of bovine serum albumin in PBS and probed overnight in 4 °C with Rabbit polyclonal Anti-Collagen I antibody (Abcam, ab34710, 1:300) or Rabbit polyclonal Anti-Collagen III antibody (Abcam, ab7778, 1:150) followed by secondary Alexa Fluor 594- conjugated Donkey Anti- Rabbit antibody (1:150, Jackson ImmunoResearch, 1 h, rt). The nuclei were visualized with DAPI staining (20 ng/mL of DAPI solution for 4 min, RT). The result was evaluated with fluorescence microscopy (Olympus IX51 and CellSens™ Microscope Imaging Software). The expression of collagens (type I and III) was measured as the area of specific fluorescence per cell [μm^2^]. At least 900 cells per well were analyzed from 10 randomly selected fields of view. Immunocytochemistry was performed on hASCs from two different donors.

### Western blot (WB)

Human ASCs were cultivated with or without BMP-12 (100 ng/ml) for 7 days on Ø 100 mm. culture dishes. Collected cell pellets were lysed with RIPA buffer (50 mM Tris, pH 7.5, 150 mM NaCl, 1 mM EDTA, 1% NP-40, 0.25% Na-deoxycholate, and 1 mM PMSF) supplemented with protease inhibitor cocktail and phosphatase inhibitor cocktail (Sigma-Aldrich) for 30 min at 4^∘^C in order to isolate protein extracts. Lysates were cleared for 20 min at 14000 rpm, and supernatants were collected. The total protein concentration was determined using Bio-Rad protein assay dye reagent according to the producer’s instructions (Bio-Rad Laboratories Inc., Hercules, CA, USA). Proteins (35 μg of total protein per well) were resolved by SDS-PAGE and transferred onto PVDF membrane (Sigma-Aldrich). For immunostaining membranes were blocked with 5% nonfat dry milk in TBS (20 mM Tris-HCl, 500 mM NaCl) containing 0.5% Tween20. The membranes were incubated with Rabbit polyclonal Anti-Collagen I antibody (Abcam, ab34710, 1:500) or Rabbit polyclonal Anti-Collagen III antibody (Abcam, ab7778, 1:1000) or Rabbit polyclonal Anti-Mohawk antibody (LSBio, aa46-75, 1:1000) or Goat polyclonal Anti-Actin (Santa Cruz Biotechnology, C-11, sc1615, 1:1000) primary antibodies. Next the blots were washed three times for 15 min and incubated with appropriate secondary antibodies conjugated with IR fluorophores: IRDye 680 or IRDye 800 CW (purchased from LICOR Biosciences; Lincoln, NE, USA) at 1:5000 dilution. Odyssey Infrared Imaging System (LI-COR Biosciences) was used to analyze the protein expression. Scan resolution of the instrument was set at 169 μm and the intensity at 5. Quantification of the integrated optical density (IOD) was performed with the analysis software provided with the Odyssey scanner (LI-COR Biosciences). Immunoblot analysis for cells from each donor was performed on samples from three independent electrophoreses. For the purpose of publication the color immunoblot images were converted into black and white images in the Odyssey software.

### Mixed lymphocyte reaction (MLR)

Ten milliliters of venous blood was collected in heparinized tubes from healthy blood donors after obtaining informed consent. Separation of peripheral blood mononuclear cells (PBMCs) was performed within 2 h of withdrawal of blood. Blood samples were taken into preservative-free heparin (20 units/ml) tubes, and PBMCs were isolated by centrifugation on Histopaque-1077 (Sigma-Aldrich) of the blood diluted 1:1 with Sodium Chloride 0.9% (0.9% NaCl, Fresenius Kabi). PBMCs were taken up in Parker medium (Biomed) supplemented with 2 mM L-glutamine (Sigma-Aldrich), 0.1 mg/ml gentamycin (KRKA), β-mercaptoethanol (Sigma), 0.23% Hepes (Sigma) and 10% fetal bovine serum (FBS, Gibco). Half of the isolated PBMCs were inactivated by gamma-irradiation for 90 min.

hASCs after 7 days culture with BMP-12 were collected and seeded onto 96-well flat-bottom plate (Greiner) in a concentration of 0.8 × 10^4^/well. Each time, cells from the same donor cultured in parallel without BMP-12 were seeded on the same plate in the identical scheme. Cells were left overnight to attach. For the MLR, 2 × 10^5^ PBMCs (1 × 10^5^ cells/well from a first donor and 1 × 10^5^ cells/well from the second donor) were co-seeded with hASCs in the following combinations: XX_ir_, YY_ir_, XY_ir_, YX_ir_ (X – first donor’s PBMCs, X_ir_ – irradiated first donor’s PBMCs, Y – second donor’s PBMCs, Y_ir_ – irradiated second donor’s PBMCs). PBMC cultures without hASCs were used as controls. Cells were cultured for 5 days at 37 °C in a humidified atmosphere with 5% CO_2_. After 5 days cells were pulsed with 1 μCi/well of 3H-thymidine (113 Ci/nmol, NEN) for the last 18 h of the incubation and harvested with an automated cell harvester (Skatron). The amount of 3H-thymidine incorporated into the cells was measured using a Wallac Microbeta scintillation counter (Wallac), giving the level of radioactivity as ‘Corrected Counts per Minute’ (CCPM). For this experiments hASCs from 5 different donors were used (*n* = 5), but the influence of hASCs from each donor was tested on PBMCs from two different blood donors (X and Y). Therefore, 10 separate experiments were performed, each in triplicate.

### Cell proliferation assay

Cell proliferation assay was conducted using a colorimetric BrdU proliferation ELISA immunoassay (Roche). hASCs after 7 days culture with or without BMP-12 (50 or 100 ng/ml) were collected and seeded onto 96-well flat-bottom plate in a concentration of 0.8 × 10^4^/well in experimental or control medium respectively. The cells were allowed to grow at 37 °C and 5% CO2 for the next 36 h. Afterwards, BrdU labeling reagent was added to each well. Then, the cells were further incubated for 12 h, and the pyrimidine analogue BrdU was incorporated in place of thymidine into the DNA in proliferating cells. Next, the immunostaining was performed according to manufacturer instructions. The absorbance was measured at wavelength 450 nm (BioTek PowerWave XS). Experiments were performed in triplicates for each sample.

### Cell migration assay

The migration assay was performed using trans-well inserts with 8 μm pore membrane (BD Biosciences, San Jose, CA, USA). The wells of the 24-well glass- bottom plates (SensoPlate, Grainer) was filled with 1 ml of different culture medium: standard GM, GM containing 50 ng/mL BMP-12, or GM containing 100 ng/mL BMP-12. Human ASCs after 7 days culture with or without BMP-12 were seeded to the upper compartment of the cell culture inserts at a density 1.5 × 10^4^ cells per insert and then inserts were placed into the proper wells. To allow cell migration from the inserts to the wells, plates were incubated at 37 °C and 5% CO_2_ for the next 72 h. Afterwards, medium was removed and the inserts were peeled off the cells that migrated to the bottom side of the membrane using 0.25% tripsin- EDTA solution (Sigma). After 24 h, when the cells adhere to the bottom of the wells, hASCs were fixed with 70% methanol and the nuclei were visualized with DAPI staining (20 ng/mL of DAPI solution for 4 min). Results were assessed with a cell imaging multi-mode microplate reader Cytation™ 3 (BioTek) which allowed to specify the number of objects giving a signal of blue fluorescence (DAPI, wavelength: 377–477 nm). Experiments were performed in duplicates for each sample.

### hASCs viability under oxidative stress in vitro

hASCs after 7 days culture with or without BMP-12 (100 ng/ml) were collected and seeded onto 96-well flat-bottom plate in a concentration of 0.8 × 10^4^/well in respective experimental or control medium. Cells were left to attach and after 24 h were exposed to oxidative stress by adding a medium with hydrogen peroxide in increasing concentrations (0, 750, 1000, 1500 μM). After 22 h of incubation, 20 μl of 3-(4,5-dimethylthiazol-2-yl)-2,5-diphenyl tetrazolium bromide (MTT, Sigma-Aldrich) at the concentration of 5 mg/ml in PBS were added and incubated for another 2 h. The medium was discarded and 100 μl of DMSO was added. The plate was shaken for 10 min using microplate shaker. Absorbance, corresponding to the mitochondrial dehydrogenases activity of viable cells was measured colorimetrically at wavelength 570 nm (BioTek PowerWave XS).

### Secretory activity

Like in other experiments, hASC were cultured with or without BMP-12 (100 ng/ml) for 7 days. For the last 48 h the serum was withdrawn from the medium and cells were incubated with DMEM-LG supplemented with 4% bovine serum albumin and antibiotics (1%) with or without BMP-12 respectively. At the end of incubation period, the supernatants were collected and frozen in -80 °C. Luminex multiplex assays (Procarta) were used for measuring concentration of the following molecules: IL-6, TNF-α, IL-10, EGF, VEGF, MMP-1, MMP-8 and MMP-13. TGF-β1 concentration was evaluated with ELISA kit (R&D) according to the manufacturer’s instruction. All experiments were done in duplicates.

### Statistical analysis

For data analysis STATISTICA software (StatSoft®Polska) was used. Data are presented as medians, quartiles and min-max or means ± SEM. Differences between groups were analyzed by non-parametric Wilcoxon test for related data or U Mann-Whitney if non-related data were compared. Student *T*-test was used if in compared groups consisted of at least 10 values with normal distribution confirmed by Shapiro-Wilk test. The *p* value less than 0.05 was considered as statistically significant.

## Results

**Fig. 1:**
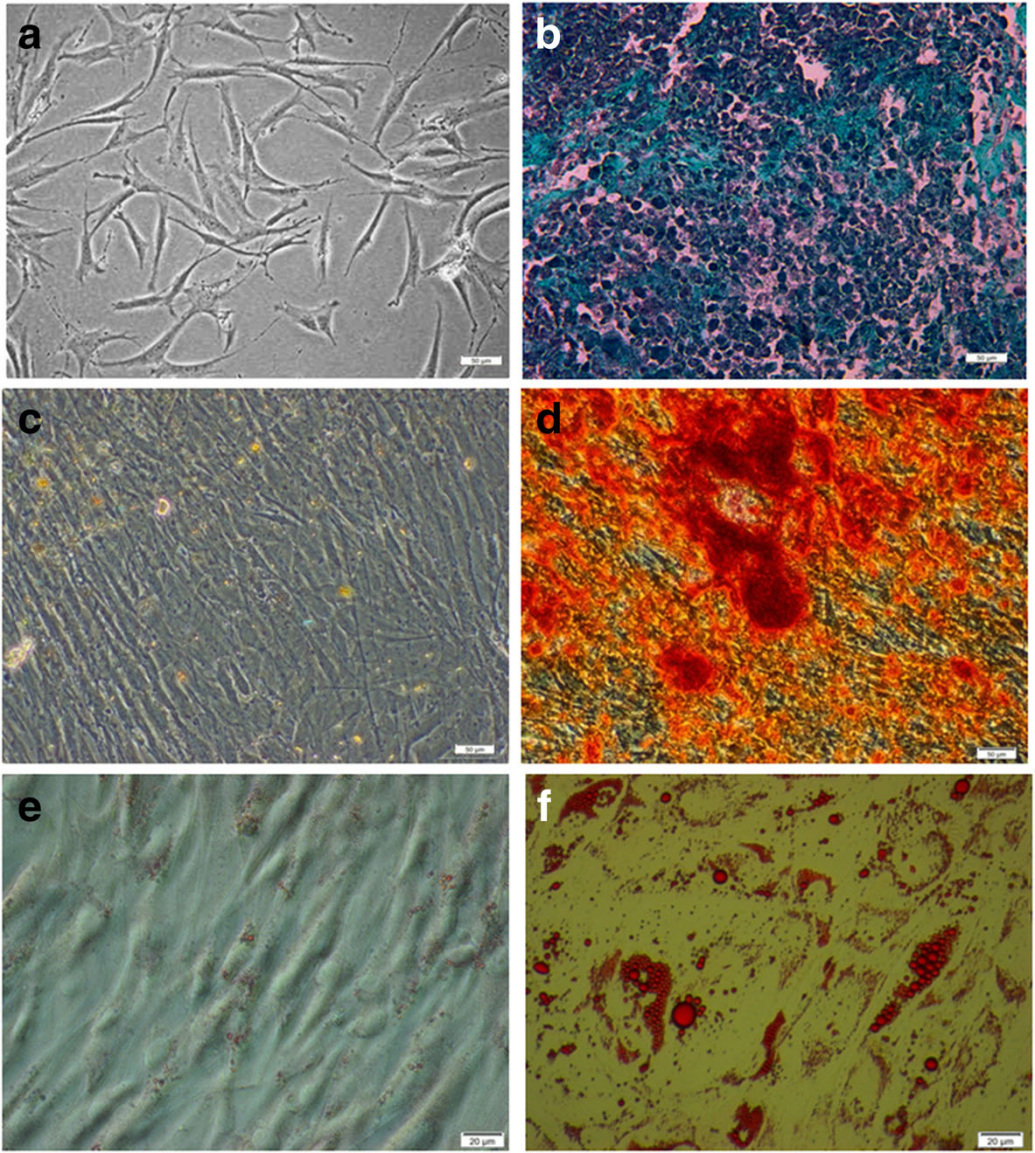
Human ASCs differentiation potential. light microscopy: **a** morphology of undifferentiated hASCs, **b** hASCs after chondrogenic differentiation, chondropellet stained with Masson’s Trichrome method, collagen deposits are *blue*; **c**, **d** Alizarin Red staining (calcium deposits are *red*) of hASCs cultured in standard (**c**) or osteogenic medium (**d**); Oil Red O staining (lipid droplets are *red*) of hASCs cultured in standard (**e**) and adipogenic (**f**) medium. Representative data from one donor are presented. Scale bars: 50 μm (**a**, **b**, **c**, **d**); 20 μm (**e**, **f**)

Cells were successfully isolated from 6 donors. All populations were able to form colonies and adhere to plastic surface. To characterize hASCs populations the surface antigen profiles were examined. Flow cytometry analysis demonstrated the expression of CD29 (median 93% of positive cells), CD73 (median 96%), CD90 (median 96%) and CD105 (median 87%) and revealed no (median less than 1%) expression of hematopoietic cell lines marker-CD45. Human ASCs showed the ability to differentiate into adipocytes, osteocytes and chondrocytes which was confirmed by specific staining. The representative effect of differentiation procedure are presented in Fig. 1.

### The effect of BMP-12 on tenogenic differentiation of hASCs

**Fig. 2:**
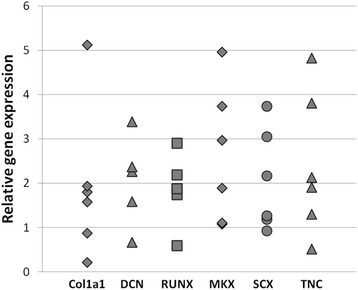
BMP-12 treatment activates tenogenic pathway in hASCs. Real time PCR in human adipose stem cells (hASCs) treated or not with 100 ng/ml BMP-12 for 7 days. Results presented as fold of change in relation to the internal control samples (cells from the same donor cultured in parallel in standard growth medium). hASCs obtained from 6 independent donors were used. Col1a1- Collagen, type I, alpha 1; DCN- Decorin; RUNX- Runt-related transcription factor 2; MKX- Mohawk homeobox; SCX- Scleraxis; TNC- Tenascin; GAPDH gene was used for normalization

**Fig. 3:**
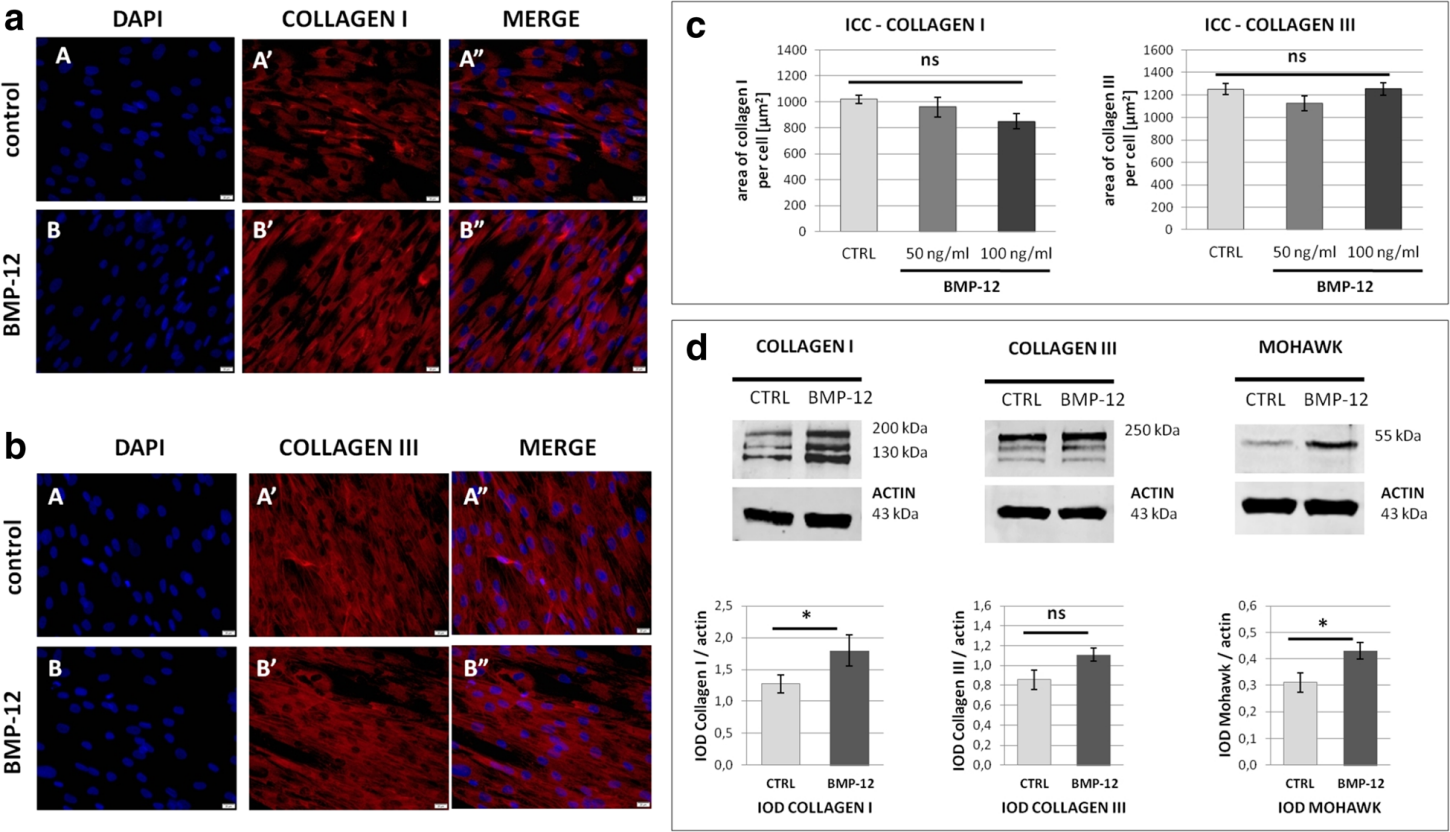
The effect of BMP-12 treatment on tendon associated proteins expression in hASCs. **a**, **b** Immunocytochemistry (ICC). The expression of collagen I (**a**) and collagen III (**b**) (were stained in *red*) in hASCs cultured for 7 days in standard medium (control, *upper panel*), or in medium supplemented with 100 ng/ml BMP-12 (*lower panel*). Nuclei were stained with DAPI (*blue*). All pictures present cells from the same donor and the same experiment. Scale bars - 20 μm. **c** Quantification of ICC results. The collagen I and III area per cell was calculated [μm^2^] after 5 days culture in medium with or without BMP-12 (50 or 100 ng/ml). Data obtained from 2 donors. For each treatment/protein/donor at least 900 cells were subjected to analysis. Kruskal–Wallis one-way analysis of variance detected no statistical differences between groups (ns). **d** Western blot analysis for collagen I, collagen III and mohawk. Expression of actin was used as a loading control. Graphs below representative bands demonstrate densitometric analysis (normalized to IOD of corresponding actin). The results presented as means ± SEM from three donors (at least three separate electrophoreses per donor performed and analyzed). Data analyzed using non-parametric Wilcoxon test in comparison to internal control (BMP-12 untreated cells from the same donor and electrophoresis).* - *p* < 0.05

Treatment of hASCs with BMP-12 for 7 days resulted in an increase of *SCLERAXIS* and *MOHAWK* genes expression (*p* < 0.05 for both transcription factors). The mean change in relative expression amounted respectively 2.05 and 2.65 folds in comparison to control, untreated samples. BMP-12 treatment induced also up-regulation of osteogenic factor- *RUNX2* (mean change in relative expression was 1.87 fold, *p* < 0.05). Although the changes of dCt values for all analyzed transcription factors were statistically significant, it is worth to stress that the response of cells from independent donors was diverse what illustrates Fig. 2. The gene expression of *COLL1α1, TNC* and *DCN* did not differ significantly. Immunocytochemical method revealed that untreated hASCs expressed both collagen type I and type III in both tested populations (Fig. 3a, b). Quantification of ICC results demonstrated no significant differences between groups (CTRL vs BMP-12 treated cells, Fig. 3c). To verify those results, Western blot analysis was additionally performed and this technique indicated that BMP-12 treatment (100 ng/ml, 7 days) increased expression of collagen type I and mohawk in hASCs (mean 1.8 and 1.5 folds, respectively, both *p* < 0.05). The expression of collagen type III was also increased by mean 1.4 fold, but this change was not statistically significant (Fig. 3d).

### BMP-12 do not affect proliferation rate and migration capacity of hASCs

**Fig. 4:**
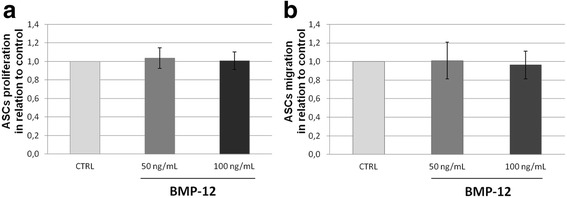
BMP-12 treatment does not affect neither hASCs proliferation nor their migration. **a** Proliferation activity of hASCs determined using a colorimetric BrdU proliferation ELISA immunoassay after culture in the presence of BMP-12. Cells from the same donor cultured in parallel in standard growth medium were used as a control. **b** Trans-well migration test (inserts with pore 8 μm). hASCs migration evaluated after 7 days culture with or without BMP-12. Data presented as relative values to the internal control (untreated hASCs from the same, respective donor); the number of donors *n* = 6, tests performed in triplicates (**a**) or in duplicates (**b**). No statistically significant differences were detected

There were no significant differences between proliferative rate of hASCs treated with BMP-12 in comparison to the non-treated cells regardless of the applied BMP-12 dose (Fig. 4a). Likewise, migration capacity of BMP-12-treated cells was not significantly different from those of internal control (Fig. 4b). Those results were consistent among all tested populations (*n* = 6). Combined results from all donors are presented in Fig. 4.

### BMP-12 significantly increases secretion of VEGF, MMP1, MMP8 and IL6 in hASCs

**Fig. 5:**
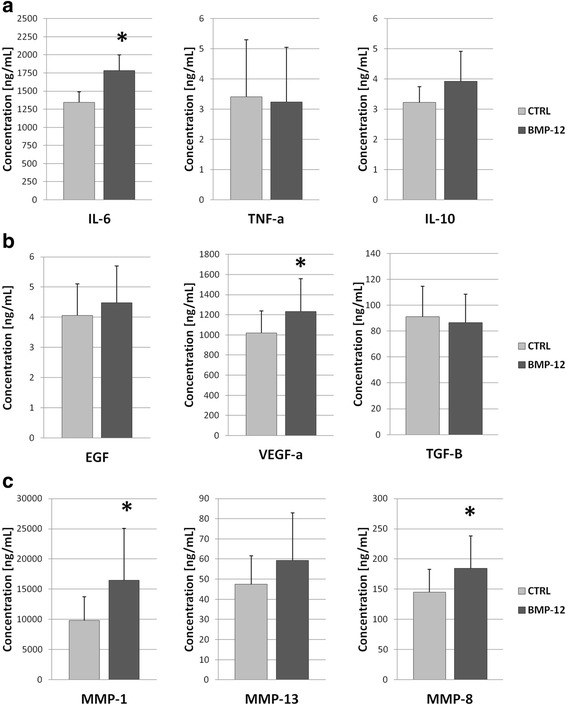
BMP-12 treatment affects secretory activity of hASCs. Cytokines (**a**), growth factors (**b**) and metalloproteinases (**c**) determined in serum free supernatants collected from above hASCs treated or not with 100 ng/ml BMP-12 (7 days, supernatants collected from last 48 h). Data presented as mean +/- SEM. Results analyzed using non-parametric Wilcoxon test in comparison to internal control (BMP-12 untreated cells from the same donor).* - *p* < 0.05. All tests were performed in duplicates; *n* = 6 donors

BMP-12 (100 ng/ml) treatment (7 days) affected significantly hASCs secretory activity in regard to IL-6, VEGF, MMP-1 and MMP-8. All concentrations were analyzed using non-parametric Wilcoxon test for related values - treated sample in comparison to the control sample (untreated cells from the same donor). The mean increase of IL-6 concentration in supernatants after BMP-12 treatment amounted 37% (*p* = 0.027). The mean increase of pro-angiogenic VEGF after BMP-12 treatment was 17% (*p* = 0.046), whereas the mean increase of MMP-1 and MMP-8 concentrations were 41% (*p* = 0.027) and 21% (*p* = 0.027) respectively (Fig. 5). In case of IL-6 and MMP-8 an increase in secretion after BMP-12 treatment was detected in all analyzed populations (*n* = 6), whereas in case of VEGF and MMP-1 an increase in secretion after BMP-12 treatment was noted in 5/6 tested populations. As both proliferation rate (BrdU assay) and metabolic activity (MTT assay) was no affected by BMP-12 treatment in the same cells, we conclude that noted differences were the effect of BMP-12 action on analyzed hASCs. No significant changes in secretion of EGF, IL-10, TGF-b I MMP-13 were observed. Combined results from all donors are presented in Fig. 5.

### BMP-12 significantly reduces the ability of hASCs to suppress lymphocyte proliferation

**Fig. 6:**
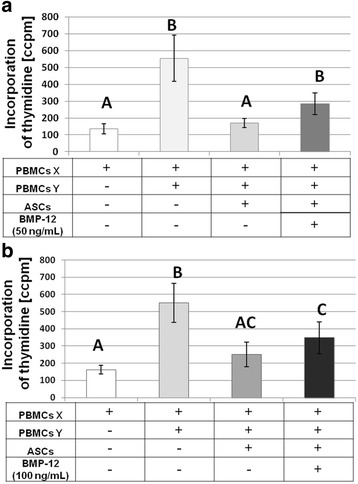
BMP-12 treatment impairs immunomodulatory properties of hASCs. The effect of hASCs treated or not with BMP-12 (**a**. 50 ng/ml and **b**. 100 ng/ml) for 7 days on proliferation of PBMCs (peripheral blood mononuclear cells) in Mixed Lymphocytes Reaction (MLR). PBMCs from two independent donors X and Y were used for each experiment. Isolated PBMCs were stimulated in autologous or allogeneic manner (by addition of irradiated PBMCs from the same or second donor respectively). Mixture was cultured with or without addition of hASCs (treated with BMP-12 or not). +/- indicates the presence of a given component. Data presented as means and SEM. Results analyzed with Student *T* test or non-parametric Wilcoxon test depending on distribution. Letters above bars indicate statistical differences: groups sharing the same letter do not differ significantly (*p* < 0.05). Number of hASCs donors *n* = 5, each hASC population tested with PBMCs from two different donors (X and Y). The amount of 3H-thymidine incorporated into the cells giving the level of radioactivity as ccpm – corrected counts per minute, tests performed in triplicates

ASCs are known for their immunomodulatory properties. Mixed lymphocyte reactions (MLR) were performed to determine whether treatment of hASCs with BMP-12 changes theirs in vitro immunosuppressive activity (Fig. 6). As expected, the addition of allogeneic irradiated PBMCs from donor Y to PBMCs from donor X resulted in a massive increase in lymphocytes proliferation (*p* < 0.01 in comparison to auto-stimulated cells). The addition of hASCs to the mixture of PBMCs (X + Y_ir_ or opposite) significantly inhibited allo-activated lymphocytes proliferation (*p* = 0.016) to the level which was not significantly different than in control lymphocytes (auto-stimulated). This inhibition was significantly decreased when hASCs were previously exposed to 50 ng/ml BMP-12 for 7 days (*p* = 0.037 between X + Y_ir_ + hASCs and X + Y_ir_ + hASCs + BMP-12 (50), a decrease observed in 8/10 observations). Similar effect (a decrease in inhibition in 8/10 observations) was noted in reaction with cells treated with 100 ng/ml BMP-12, but the difference between X + Y_ir_ + hASCs and X + Y_ir_ + hASCs + BMP-12(100) did not reach statistical significance. In reactions, where hASCs were treated with BMP-12 (both 50 and 100 ng/ml) lymphocyte proliferation became significantly higher than in control auto-stimulated ones. Figure 6 presents combined data from 5 hASCs donors (each paired with 2 different PBMCs donors, giving together 10 observations).

### BMP-12 do not change the susceptibility of hASCs to oxidative stress

**Fig. 7:**
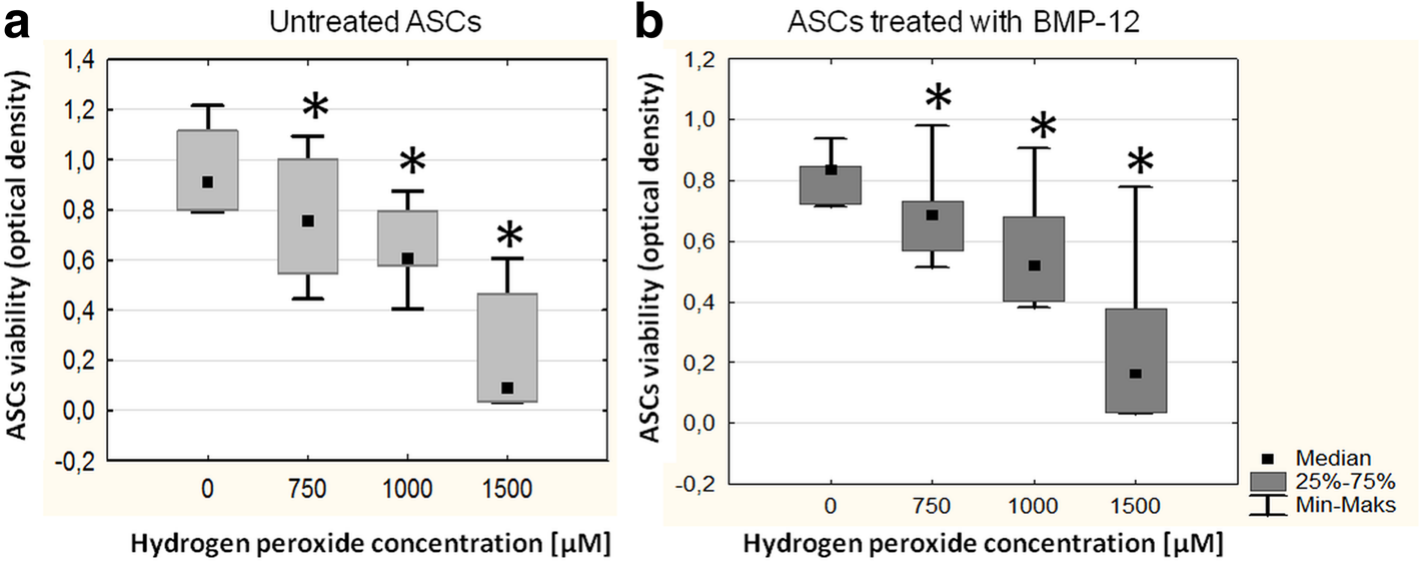
BMP-12 treatment does not affect susceptibility of hASCs to oxidative stress in vitro. MTT test. Metabolic activity (related to viability) of hASCs after exposure to growing concentrations of hydrogen peroxide (24 h exposure). **a** control hASCs (cells from the same donor cultured without BMP-12); **b** hASCs after BMP-12 treatment (100 ng/ml, 7 days before exposure to H_2_O_2_). Data presented as medians, quartiles and min-max. Results analyzed using non-parametric Wilcoxon test in comparison to H_2_O_2_ untreated sample.* - *p* < 0.05. All tests were performed in triplicates; number of hASCs donors *n* = 6

The effect of oxidative stress on cells viability was determined in MTT test. In both control and BMP-12 treated cells there were significant differences in viability after 24 h exposure to growing concentrations of hydrogen peroxide compared to H_2_O_2_ untreated sample. Differences were analyzed sing non-parametric Wilcoxon test. BMP-12 treatment had no effect on hASCs resistance to oxidative stress - in both, control and BMP-12 exposed cells, 750 μM of H_2_O_2_ was required to significantly (*p* < 0.05) impair cell viability (Fig. 7). Additionally, the comparison of BMP-12 untreated and treated hASCs viability was performed within certain level of oxidative stress (750, 1000, 1500 μM). The analysis was performed using U Mann Whitney test. No significant differences were detected, the results were consistent among tested populations (*n* = 6) and in combined version are presented in Fig. 7.

## Discussion

Mesenchymal stem cells are considered as a candidate population for cell therapy in many different applications including orthopedic disorders like non-union bone fractures, osteoarthritis or more recently, tendon injuries [17]. In orthopedics, cell administration is usually local (not systemic) and it is excepted that cellular transplantation will improve regeneration of target tissue. Nevertheless, there are at least three different mechanisms by which MSCs potentially ameliorate tissue healing: 1) via differentiating, 2) via paracrine activation of endogenous progenitor cells and promoting angiogenesis, 3) via controlling inflammatory response and directing macrophages into M2 phenotype. The first, most obvious and primarily described mechanism is acting through differentiating into the target tissue cells. The capacity of MSCs (regardless of their source) to enter osteogenic and chondrogenic pathway is undoubtful and became one of identification requirement [18]. The ability to differentiate into tenocytes is not so well documented and not so easy to proof. Presented herein results demonstrate that BMP-12 induced up-regulation of genes for main transcription factors associated with tenogenesis. Previously, BMP-12 was shown to activate tenogenic pathway in rat and human bone marrow derived MSCs [12, 13]. In regard to adipose derived stem cells, the effect of BMP-12 treatment was studied on canine [16] and human cells [19]. It is important to stress that it is difficult to clearly state if a cell (especially in 2D culture) is already a tenocyte or not. There is no unequivocal method to recognize completed tenogenic differentiation as it is a case in osteogenesis (extracellular calcium deposits formation) or adipogenesis (intracellular lipid droplets). Tenocytes produce large amount of ECM composed predominantly of collagen type I which is the most abundant ECM component in the whole organism and many cell types including undifferentiated MSCs express this protein. Other ECM molecules associated with tenocytes are collagen type III, decorin, tenascin C, tenomodulin, however, none of them is exclusively expressed by tenocytes. Herein, it was confirmed that undifferentiated hASCs produce both key tendon associated collagens: type I and III (Fig. 3a,b). The effect of BMP-12 on protein expression of collagen type I and III was analyzed in this study with two methods - quantified ICC and WB. In regard to collagen type I expression the results were not consistent - ICC revealed lack of significant difference between control and treated cells whereas WB demonstrated statistically significant increase of collagen type I after BMP-12 treatment (Fig. 3d). As all cells expressed collagen type I, the ICC analysis could reflect rather cell size than the actual protein expression. Therefore, WB in this case seems to be a superior method. Other factors identified as key tenogenic markers are transcription factors: scleraxis and mohawk [12, 20], nonetheless those proteins are also expressed by undifferentiated MSCs [16]. Therefore, an in vitro evaluation of tenogenesis in two dimensional culture is always based only on relative changes in set of molecules expression. In our study we noticed an increase of tenogenic transcription factors in hASCs after BMP-12 treatment what is in agreement with previous studies performed on MSCs from other sources or species [12, 16, 19]. One previous research on human ASCs [19] showed increased expression of genes after BMP-12 treatment, but only an increase in *DECORIN* expression was statistically significant. In the mentioned study tenogenesis inducing medium contained reduced serum concentration (1% FBS) compared to control growth medium (10% FBS). For this reason cannot be unambiguously assess whether the decrease in the serum concentration can induce tenogenic path without the addition of BMP-12. In the present study, the differences in tenogenic transcription factors expression achieved statistical significance, however, the mean change (2-fold increase in case of *SCLERAXIS*) was very similar to results presented by Stanco et al. [19]. Presented herein results demonstrate also up-regulation of *MOHAWK* after BMP-12 treatment what is in agreement with data reported by Otabe et al [12]. In our study we further confirmed increased MOHAWK expression in response to BMP-12 by Western blot analysis. Nevertheless, in cells treated with BMP-12 we also noticed up-regulation of osteogenic transcription factor *RUNX2* (*p* < 0.05). Although it was demonstrated that BMP-12,-13 possess significantly lower osteogenic activity than classical bone associated BMP-2 [21], certain level of osteogenic BMP-12 potential was presented in other studies [16]. Ectopic tissue calcification is one of the concerns associated with MSC-based therapy. Moreover, tenogenic and osteogenic intracellular signaling pathways are very similar. Therefore, a risk of unfavorable differentiation in case of BMP-12 treatment should be further investigated.

Another described mechanism by which MSC can improve tissue regeneration is to promote angiogenesis through secreted molecules. The key mediators of new blood vessel formation are vascular endothelial growth factors. Mesenchymal stem cells are known to be a stable source of VEGF [22]. Presented herein results show that BMP-12 treatment significantly enhances secretion of VEGF by hASCs. It was previously demonstrated that VEGF secretion can be a crucial mechanism of MSCs action in nerve repair [23], wound healing [24], myocardium regeneration [25] or recovery from acute kidney injury repair [26]. In regard to tendon associated disorders, the role of VEGF is not so clear. In chronic tendinopathies, neovascularisation is perceived as unfavorable process which leads to painful neoinnervation [27]. On the other hand, in acute tendon injuries VEGF is rather a beneficial player. Its secretion was shown to be important in improvement of tendon graft maturation and biomechanical strength during anterior cruciate ligament healing after stem cell transplantation [28]. Similarly, administration of exogenous VEGF to the experimentally injured tendon enhanced healing parameters in comparison to the control, untreated animals [29, 30]. Finally, the relatively low level of endogenous VEGF mRNA following injury, supports its potentially beneficial role as exogenous modulator to optimize tendon healing and strength [31]. The relationship between BMP-12 (-13, -14) treatment and VEGF secretion has not been previously demonstrated, however, it was reported that BMP-4 significantly stimulated VEGF synthesis in osteoblast-like MC3T3-E1 cells [32]. The authors later proofed that this interaction was MAP kinase- dependent [33]. The release of VEGF by MSCs was demonstrated to be mediated by both STAT3 and p38 MAPK [34]. Generally, the basic signaling process for bone morphogenic proteins goes through Smads activation (Smad 1/5/8) [15]. Activation of this particular pathway was also confirmed specifically for BMP-12 in canine ASCs [16]. Nevertheless, it was shown by Nakashima et al. that interaction between STAT3 and Smads can occur resulting in a synergistic signaling effect [35]. This phenomenon could be one of possible explanations BMP- VEGF secretion interrelation.

Interestingly, another molecule which was herein shown to be affected by BMP-12 treatment is IL-6. This cytokine was initially described as a classical pro-inflammatory molecule because its serum concentration was found to be significantly elevated in patients with various inflammatory diseases [36]. Later, it was shown, that IL-6 is a pleiotropic cytokine which can exert both pro- and anti-inflammatory effect [37]. It is believed that the way of action depends on the signaling pathway: either it is classic signaling thought membrane bound IL-6R (anti-inflammatory effect) or trans-signaling thought soluble IL-6R which interact with gp130 protein (pro-inflammatory effect) [38]. Mesenchymal stem cells are known to be an abundant source of IL-6 [39] which was confirmed in the presented study. We additionally shown that BMP-12 treatment elevates the secretion rate of this cytokine by hASCs. Because of mentioned dual role of IL-6 in inflammatory reaction it is difficult to predict what consequences would have an increased level of IL-6 in in vivo situation after cell transfer - probably the effect will depend on local inflammatory status of a host tissue. As interleukin-6 acts intracellularly through STAT-3, it might be that elevated extracellular level of IL-6 induces STAT-3 dependent pathway in ASCs and by previously mentioned interaction with Smads can enhance VEGF secretion. It is also possible that increased level of IL-6 is related to the decreased inhibition of PBMCs proliferation by ACSs after BMP-12 treatment. It is known that IL-6 in combination with TGF-β triggers differentiation of naive Th cells towards Th17 pathway and inhibits the generation of Foxp3+ T regulatory cells induced by TGF-beta [40]. On the other hand, one of MSCs immunomodulatory mechanisms is to cause an increase in the proportion of regulatory T cells in PBMCs [41]. The TGF-β level secreted by ASCs persists unchanged and the IL-6 release is elevated under BMP-12 exposure. Therefore it is possible that more naive T cells in co-culture with BMP-12 treated ASCs differentiate toward Th17 than into T reg cells in comparison to the co-culture with untreated ASCs. This hypothesis remains to be verified. However, regardless of the mechanism, our results suggest that BMP-12 pretreatment or co-administration can be contraindicated in allogeneic ASCs (MSCs) therapies.

The key molecular feature of tendon tissue is the composition of extracellular matrix and its specific spatial arrangement. Therefore, effective remodeling of tendon ECM after injuries is particularly important. The crucial players in this process are matrix metalloproteinases, including those responsible for degradation of type I collagen which are MMP-1, MMP-8 and MMP-13 investigated in this study. All of them are released by mesenchymal stem cells irrespective of their source [42] and we demonstrated herein that BMP-12 treatment significantly elevated the secretion of MMP-1 and MMP-8 by human adipose derived stem cells. MMPs were shown to have role in physiological tendon healing [43], but on the other hand it is suggested they are involved in pathogenesis of chronic tendinopathies i.e., rotator cuff disease [44]. Tendon tissue is constantly turned over with higher rates at sites exposed to high level strain. It is believed the lost of balance between MMPs and their inhibitors can predispose to a chronic tendinopathies. Among collagenases, MMP-1 and MMP-13 were shown to be up-regulated in rotator cuff tear [45, 46]. Therefore, our results showing increased release of MMP-1 by hASCs after BMP-12 treatment suggest that using this protein in combination with cell therapy can be not beneficial in chronic tendinopathies.

Potential use of BMP-12 in combination with MSCs (ASCs) therapy required examination of BMP-12 effect on basic cellular activities like proliferation and migration. BMPs belongs to TGF-β superfamily which generally are known to inhibit cell proliferation [47]. However, it can be concluded from different studies that effect of BMPs on cell proliferation vary between both cell types and different BMPs. For example, BMP-3 were shown to promotes MSCs proliferation [48] whereas BMP-9 inhibited bFGF induced proliferation of endothelial cells [49]. Our results demonstrated that BMP-12 (50 or 100 ng/ml) had no influence on hASCs division rate what is in agreement with data presented by Haddad-Weber et al. [50]. Also, BMP-14 (GDF-5) was demonstrated to not affect human MSCs proliferation [51]. This finding is in accordance with the weak effect of BMP-12 on hASCs tenogenic differentiation which was observed in this study. It is generally accepted that the process of differentiation into relatively stable tissues like muscle, bone or tendon is associated with the significant decrease of proliferation in progenitor cells. In regard to cell migration, the effect of BMPs is not well recognized. In the discussed study, we demonstrated that BMP-12 had no effect on hASCs migration capacity what seems to be beneficial in term of potential local cell administration.

## Conclusions

In summary, MSC-based therapy is a promising treatment method in many orthopedic conditions including different tendon disorders. One of proposed approach in stem cell transplantation is to pre-differentiate cells in vitro before injection to activate certain pathways and facilitate direct regeneration of disabled tissue. In the present study we demonstrated that BMP-12 induces tenogenic pathway in human hASCs as it up-regulates key tenogenic transcription factors (*SCLERAXIS* and *MOHAWK*). However, BMP-12 treatment affected not only differentiation process in hASCs. It caused also significant changes in secretory activity of treated cells and impaired their immunomodulatory properties. The enhanced secretion of VEGF and collagenases could possibly improve regeneration process in acute tendon injuries, but in chronic tendinopathies, neoangiogenesis and MMP over-activity are rather markers of pathologic processes. Therefore, our results suggest that BMP-12 could be a candidate for cell pretreatment in cases of acute tendon injuries, but not in chronic tendinopathies.

## Additional file

Additional file 1: Study design scheme. Diagram shows the steps in the course of the study. After 7 days of culture with or without BMP-12 supernatants were collected and cells harvested. Depending on the requirements of each experiment cells were plated in 96- or 24-well plate. For analysis of gene expression RNA was isolated immediately after the end of treatment. (PDF 686 kb)

## Abbreviations

ASCs: Adipose stem cells
BMP: Bone morphogenic protein
GDF: Growth and differentation factor
ICC: Immunocytochemistry
MLR: Mixed lymphocyte reaction
MSCs: Mesenchymal stem cells
PBMCs: Peripheral blood mononuclear cells
WB: Western blot
