# Supplementary material for: Bmp-12 activates tenogenic pathway in human adipose stem cells and affects their immunomodulatory and secretory properties
Source: BMC Cell Biol. 2017 Feb 18;18:13. doi: 10.1186/s12860-017-0129-9 (PMC5316159; doi:10.1186/s12860-017-0129-9)
Supplement: Additional file 1: — Study design scheme. Diagram shows the steps in the course of the study. After 7 days of culture with or without BMP-12 supernatants were collected and cells harvested. Depending on the requirements of each experiment cells were plated in 96- or 24-well plate. For analysis of gene expression RNA was isolated immediately after the end of treatment. (PDF 686 kb) [file 12860_2017_129_MOESM1_ESM.pdf]

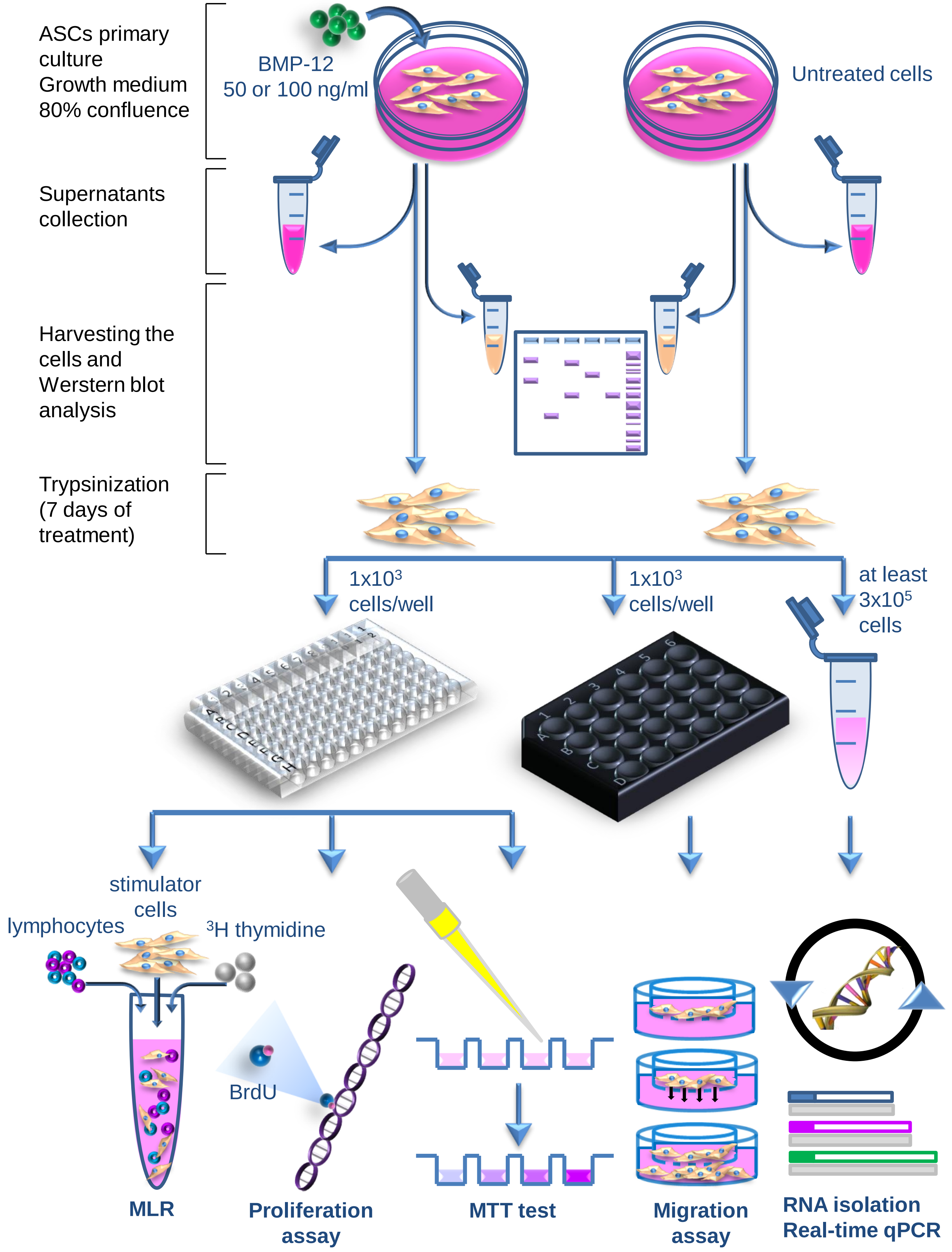
ASCs primary culture
Growth medium
80% confluence
BMP-12
50 or 100 ng/ml
Untreated cells
Supernatants collection
Harvesting the cells and Werstern blot analysis
Trypsinization (7 days of treatment)
1x10³ cells/well
1x10³ cells/well
at least 3x10⁵ cells
stimulator cells
lymphocytes
³H thymidine
BrdU
MLR
Proliferation assay
MTT test
Migration assay
RNA isolation
Real-time qPCR
